# Pure Air

**Published:** 1884-03

**Authors:** 


The reader has no doubt felt long ago in this narration how doubly essential to human health is the pure air of heaven, for it alone can purify the blood; it only can make blood out of the nutriment of the system. How infinitely essential, how gloriously useful is PURE AIR AND A PLENTY OF IT, in making the human frame all that it ought to be—all that it was intended to be.

But if the food be imperfect, its nutritive essence must be imperfect, and no air, however pure in quality, or in quantity large, can make a perfect blood out of it. We thus arrive at a sweeping general fact, that in order to have a perfect life-giving blood under the most favorable circumstances, the food we eat must be perfect.

The vegetables we cook must be fresh and perfect of their kind. The meats we consume must be the untainted meat of healthy animals. And both vegetables and meats should be properly and well cooked, and no more.

But to return to the new-born *tubercle.* How does it destroy the lungs? In going into an apple orchard, some trees appear to be well filled with fruit, equally distributed. Other trees have bunches of apples in patches, and by reason of varied exposure to the sun, we observe apples ripened in one spot, ripening in another, and quite green in a third. So it is, if we could see the lungs of people. In some, tubercles are thickly and equally distributed over the lungs. In others they are scattered about, a patch here, another there, a third yonder. A patch ripening in the first place; just beginning to turn in the second; while in the third, they are young and hard, and may never be different. A blackberry patch is a good and useful illustration of this point. It is the key which unlocks all the mysteries of quackery. There is a truth here, which every consumptive should understand, for there is more curative virtue in it, than in all medicine. It is wonderful how it has been lost sight of professionally. It is amazing how people won't see it. And the honest physician remains but a Cassandra still—a prophet, whose teachings, truth as they are, are wholly disregarded. But more of this in another place.

As an apple grows, it takes up more room, and soon touches its neighbor. Tubercles increase, meet, soften, and rot away together, eating up the lungs as they go. That is consumption. But what makes them grow, and what makes them soften and decay away together? The nascent crude tubercle may remain stationary for half a century; may be inappreciably hurtful; may and does remain innocuous for a life-time; may be as harmless to the system as powder is harmless, if fire is kept away. In proof of this a fact is stated—a fact of every-day occurrence in the dissecting-room. Out of fifty people, dead of other diseases than consumption, and being over forty years of age, scarcely one will be found who has not more or less tubercles in the lungs. This important fact is conclusive as to one interesting point: tubercles do not necessarily destroy life, as they may lay dormant for a life-time.

But what causes the tubercles to enlarge, soften, and rot the lungs away? Instead of writing down a long list of specifications, some of which might be omitted and many forgotten, it is of prime importance to notice one effect, instead of a hundred different causes.

Tubercles enlarge and are softened by debility of body, long protracted. Whatever then has a debilitating effect on the body, whether of a mental, moral, or physical nature, is the match which fires the magazine of life and burns it to ashes. Whatever keeps the body in a debilitated condition for weeks together, is capable of softening tubercles. If there be a great many tubercles of about the same age, as it were, any debilitating cause, acting for a comparatively short time, commences the decay, which, from the number of tubercles, soon becomes general, and the constitution fails rapidly. This is rapid consumption. It is like a spark applied to a wooden tenement which has been standing for half a century—every inch of wood is a tinder-box.

The evidence of softening tubercle is the spitting up of mouthfuls of yellow matter, which falls lumpily or heavily on the floor, with uneven edges, just as if one had been chewing a rag or piece of paper somewhat soft, and thrown it on the floor. If spit in water it sinks rapidly to the bottom, or if spit into a cup where there is but a spoonful or two of water, and the cup is tilted, the contents run rapidly from side to side. When an ulcer breaks or the lungs are decaying rapidly, the matter expectorated is not unlike thick rich cream.

A truth is about being stated, whose importance is such, that the whole civilized world should keep it in remembrance. As in a tree there may be a single cluster of apples, so in the lungs there may be but a single cluster of tubercles, and the remainder of the lungs may be perfectly sound. Or there may be two clusters or a dozen; each cluster may be a large or a small one, or they may be of various sizes. The symptoms of a ripening cluster are, first, a slight unfrequent cough; then more decided, still dry; next a little mucus comes; soon a large and free expectoration of yellowish matter is observed. If the cluster be large, this yellow matter is not brought away fast enough, and it is reabsorbed into the system. This reabsorption—this mingling of the matter of decayed lungs with the blood again—gives fever, hectic, night-sweats. As soon as the decayed matter of tubercle is removed, the patient begins to get better; the cough has disappeared in great part, if not wholly, the appetite improves, strength returns, flesh is gained, and the man may live half a century.

Whatever was done remedially at the time when the matter was about got rid of, gets the credit of having cured a man in the very last stages of consumption.

If there be two or more patches of these tubercles, another softens, as the causes of softening are applied, and the same routine is gone through, perhaps until the dozenth time, which being the last, he may live on, to die many years after, of some totally different disease; or if the constitution be not strong, the man succumbs under these repeated attacks, and passes away.

The practical uses to be made of this narration of undoubted facts are various and important: first, it is useless to take any thing without the advice of a regular physician, who must be acquainted with every constituent of the remedy, so as to know in what direction its curative agencies tend; second, do nothing which common sense, joined with professional science, does not indicate as rational and wise.

The reason of these inferences is, the wide difference between an antecedence and subsequence and cause and effect. It is clearly irrational to adopt any remedy, simply because it was applied and restoration followed its application. The scientific practitioner takes no such grounds. It is not until after repeated experiments, made under every variety of circumstances, extending through months, and seasons, and years, giving a uniform result, that he lays hold of any remedy, but once laid hold of, he never rejects it for a single nor for a dozenth failure. Such is the difference between scientific medicine and quackery, between intellect and ignorance. It is only after many a long year's trial, that the skilful practitioner can be brought to say of any remedy, "I gave this, and it cured him." The charlatan speaks thus after the first trial, his ignorance sustaining his effrontery.

Hope is the highest remedy of the soul, the most efficient for the body. This Cluster Doctrine is a true groundwork for it, in consumptive disease. Surely it is Nature's remedy, for who among a thousand does not hope to the end, in consumption? The mischief lies in not making that hopefulness the ground of practical action. The consumptive hopes but does nothing, and thus it is that by hope he lives and dies.

Some of the best medical minds in the world, men who have spent a quarter of a century in examining the lungs of the dead, state to us this important, every day fact, that few people die, after forty, who have not in the lungs, the signs of having the consumption, without ever having had the slightest suspicion of the existence of the disease, and who finally died of maladies having no approximation towards it in nature. These signs, are scars of various lengths, little excavations, or cavities or puckerings of various sizes; all very small it is true, but still showing the great fact, that decay once existed there, and that the lungs may perfectly heal after having been divided or broken, or pierced, as numerous cases bear witness in the perfect recovery of men who have been stabbed in the breast, or shot through the lungs.

The great curative principle, to which the reader's attention is specially solicited, is this: In any attack of consumption, or its repetition, the patient should hope it was the last cluster to soften, and that if he can only weather this storm, it may be the last, and life and happiness may be his, for long years to come. Too much attention can scarcely be paid to this idea, and we hope every invalid reader will sleep on it nightly, and make it the ground of active, strenuous effort for health, every succeeding day, even until life's close, for the truly brave die striving. This being their motto, they do, in these diseases often, very often, outlive the prognostications of ignorance and presumption, for it is only such who can peril a prophecy of recovery or death; they speak firmly, where the physician gives opinions with trembling on the tongue.

Another practical fact, it is difficult to refrain from mentioning here:

On the partial or complete recovery from an attack of consumption, do not, as you value life, intermit a single possible effort for maintaining the highest possible degree of health; keep it up, until a habit of health is established, and even then, until the close of life, make it your study to live rationally, apportioning your eating to your exercise, as true wisdom dictates.

The symptoms of consumption have been described in a general manner. It is purposed under this head to speak of the far-off symptoms, which, if promptly treated, may eventuate in cure, with as much certainty as belongs to ordinary diseases. It is scarcely to be hoped that any attention will be paid to these, yet a book on this subject could not have claim to completeness of history without discoursing something on this head. It is certainly desirable, for it is highly practical, capable as it is, of making Consumption of the Lungs a manageable disease.

But it is sadly feared, that it is to be the consummation of future centuries.

It is with consumption as it is with cholera, easily manageable in its first stages; in its last, utterly incurable. All men looked with horror on the Asiatic curse when it first visited our shores. When it was first described as sweeping the world with death, it was represented to be as instantaneous as a plague or palsy, and without one single warning note, hurrying multitudes to the grave; and yet on more minute and scientific inquiry, it is an established fact, that cholera, when attended to, in its premonitory stages, is an easily manageable disease, and is now shorn of half its horrors.

So of consumption, if we took note of its far off symptoms, and would then enter upon a course of life wisely energetic, it becomes one of the most manageable of diseases. The important practical inquiry then arises. what are the earliest and most invariable symptoms of common consumption of the lungs?

